# Plant controls over tropical wetland nitrous oxide dynamics: a review

**DOI:** 10.1007/s42965-025-00407-7

**Published:** 2025-12-08

**Authors:** Nicholas T. Girkin, Hannah V. Cooper, Scott J. Davidson, Vincent Gauci

**Affiliations:** 1 School of Biosciences, University of Nottingham, Nottingham, UK; 2 Département des sciences biologiques, Université du Québec à Montréal, Montréal, Canada; 3 School of Geography, Earth and Environmental Sciences, University of Birmingham, Birmingham, UK

**Keywords:** Denitrification, Land use change, Nitrification, Nitrogen, Tropical peatlands

## Abstract

Tropical wetlands are an important global source of greenhouse gas emissions, including nitrous oxide, a potent and long-lasting greenhouse gas. Tropical wetland ecosystems can be highly heterogeneous, featuring a variety of vegetation types, from grasses through to palms and mangroves. While soil conditions (particularly soil moisture and pH) are essential for determining the formation of nitrous oxide in soils, plants have a central role in determining the balance of emissions. In this review, we summarise the importance of vegetation in regulating tropical wetland nitrous oxide dynamics. We show how a variety of plant-mediated processes can exert key controls over wetland plant–soil nitrogen transportation and transformations. Key mechanisms of plant regulation of dynamics include influencing substrate availability (carbon and nitrogen) through litter inputs, rhizodeposition, root turnover and plant nitrogen uptake, rhizosphere biology, and plant-mediated nitrous oxide transportation, all of which can vary between species and dominant vegetation types. We propose that there is a critical need to better quantify such processes across dominant wetland ecotypes, to support improved upscaling of emissions, and assess their sensitivity to future environmental change.

## Introduction

Tropical wetlands are an important potential source of global greenhouse gas emissions, including carbon dioxide (CO_2_), methane (CH_4_), and nitrous oxide (N_2_O), potentially accounting for as much as two-thirds of the latter at a global scale (D’Amelio et al. 2009). N_2_O is a potent, long-lasting greenhouse gas (GHG), approximately 300 times more powerful at driving climate warming than CO_2_ over 100 years and has caused 10% of total warming to date (Thompson et al. 2019). Concentrations have increased from 290 parts-per-billion (ppb) in 1940, to 330 ppb in 2017, and are rising annually by 0.3% (Thompson et al. 2019). Understanding the controls over N_2_O dynamics is therefore essential in recognising the future impacts of environmental change on emissions (e.g. alterations in precipitation and soil warming), and to identify potential mitigation strategies.

Estimates of tropical and sub-tropical wetlands extent range from 1.4 to 4.7 million km^2^ (Gumbricht et al. 2017). Tropical wetlands are highly heterogeneous in terms of vegetation, ranging from the largely undisturbed palm and broadleaved evergreen tree dominated peatlands of the Central Congo basin (Crezee et al. 2022), to Caribbean mangroves (Phillips et al. 1997), managed grasslands and woodlands of the Pantanal (Liengaard et al. 2014), high altitude Andean wetlands (Molina et al. 2018) Dand the extensively converted tropical peatlands of Southeast Asia (Cooper et al. 2019; Cole et al. 2022; Girkin et al. 2022). This heterogeneity results in biogeochemical processes that can vary from the micro-scale (e.g. plant root aeration and exudation), meso-scale (e.g. plant type, and physiology), to the landscape scale (e.g. wetland ecotype and hydrology). The role of differences in vegetation in determining the production and emission of N_2_O from tropical wetlands have thus far have been largely overlooked.

Understanding the role of plants in regulating emissions is important in several contexts: first, evidence suggests that under certain circumstances, for example, specific combinations of high nitrogen inputs and optimal water content, tropical wetlands of various types may be substantial but poorly quantified contributors to global N_2_O budgets (D’Amelio et al. 2009). The importance of this is underlined by the limited flux measurements that have been made to date in globally important wetland systems, including the Pantanal (Liengaard et al. 2014), and intact and degraded Southeast Asian peatlands (Cooper et al. 2019; Jovani-Sancho et al. 2023). Second, although emissions (by mass) are lower than those of CO_2_ and CH_4_, N_2_O has a substantially higher global warming potential, meaning that relatively small emissions will drive a disproportionate degree of warming (IPCC 2021). Third, climate feedbacks such as the altering of precipitation patterns and rising temperatures, may substantially alter plant productivity and inputs, and dominant vegetation types, altering N_2_O production and emissions. Fourth, exploitation of the potential of tropical wetland restoration as a nature-based solution to climate change (Strack et al. 2022; Girkin and Davidson 2024) will have implications for N_2_O budgets through direct and indirect impacts on carbon and nitrogen flows.

Although many of the fundamental ecological and biogeochemical processes are similar, data from relatively well-studied temperate and boreal wetland systems cannot be readily applied to the tropics, due to substantial differences in plant species and plant functional types, ecosystem productivity, and climate (Sjögersten et al. 2014). This hampers the further development of process-based models that can accurately scale fluxes or test their sensitivity to future environmental perturbations (Farmer et al. 2011). Understanding the role of plants is therefore important to the task of identifying local and regional emissions hotspots, develop management practices that might mitigate emissions, and to understand potential impacts from global environmental change processes, including climate impacts and land use change that will affect dominant vegetation types (Girkin and Cooper 2022). In this review, we assess the direct and indirect mechanisms by which plants and plant inputs may be regulating soil and sediment N_2_O emissions. In so doing, we identify the dominant pathways and processes underpinning production to understand potential feedbacks from ongoing global environmental change processes and aim to highlight the critical need for better quantification of the scale of tropical wetland N_2_O emissions.

## Nitrous oxide emissions from tropical wetlands

Approximately two-thirds of biological nitrogen fixation occurs in tropical wetlands (Maltby and Barker 2009). Nitrogen losses are predominantly driven by denitrification forming N_2_O and/or atmospheric nitrogen (N_2_) in a series of microbially-mediated processes. However, rates of nitrogen loss are generally much lower than inputs, making wetlands an important pool of nitrogen, with the majority stored in the organic pool in the microbial biomass, as recalcitrant organic matter, in macrophytes, and in plant litter (Reddy and DeLaune 2008; Liengaard et al. 2014). Key environmental controls over wetland soil and sediment nitrogen cycling have been elucidated, and range from soil moisture, temperature, and pH (Butterbach-Bahl et al. 2013; Girkin and Cooper 2022).

**Fig. 1:**
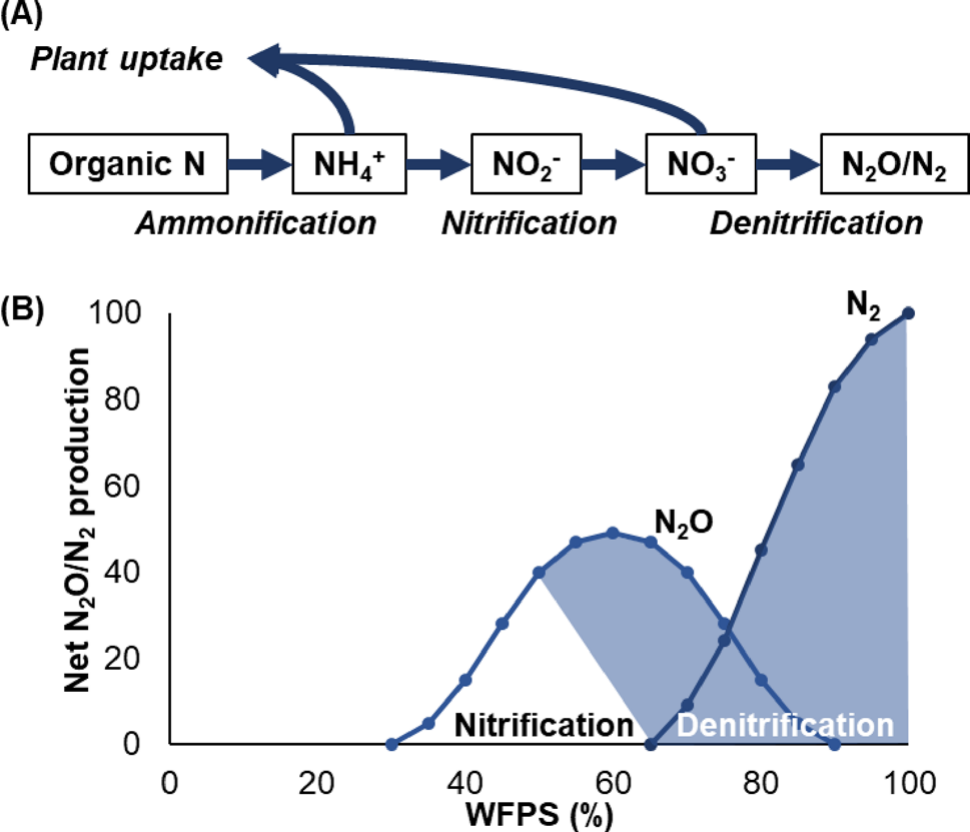
**A** Dominant nitrogen transformation pathways in wetland soils, and **B** relationship between water-filled-pore-space (WFPS) and N_2_O/N_2_ production. Shaded blue areas indicate the dominance of denitrification versus nitrification

Microbial processes drive approximately 90% of global N_2_O emissions (Butterbach-Bahl et al. 2013). Nitrification, the sequential oxidation of ammonium (NH_4_^+^) to nitrite (NO_2_^−^) and nitrate (NO_3_^−^), and denitrification, the reduction of NO_3_^−^ to N_2_O and dinitrogen (N_2_), are recognised as the dominant processes in wetland soil nitrogen dynamics (Reddy and DeLaune 2008; Girkin and Cooper 2022). While the balance of these processes is determined by substrate supply, oxygen availability, moisture, and pH, both processes can occur simultaneously in soil microsites due to differences in oxygen availability. Nitrifiers can release N_2_O at low oxygen availability when moisture content is equivalent to 60% water filled pore space (WFPS) (Fig. 1) (Bateman and Baggs 2005; Bahram et al. 2022). Similarly, denitrifiers preferentially produce N_2_O under low oxygen conditions (Molina et al. 2018). The proportion of N_2_O as the end of product of denitrification increases at lower pH and may thus represent the dominant gaseous nitrogen production pathway in tropical wetlands (Reddy and DeLaune 2008). Other processes may also contribute to emissions, but the balance of pathways is largely unknown. For example, in tropical peatlands, dissimilatory nitrate reduction can occur under NO_3_^−^ limiting conditions (Espenberg et al. 2018), as can anaerobic ammonium oxidation (ANAMOX) (Hu et al. 2011). Flooded wetland areas often emit little N_2_O and can even be periodic N_2_O sinks, while drier non-ponded areas may represent more substantial sources (Tangen and Bansal 2022).

**Fig. 2:**
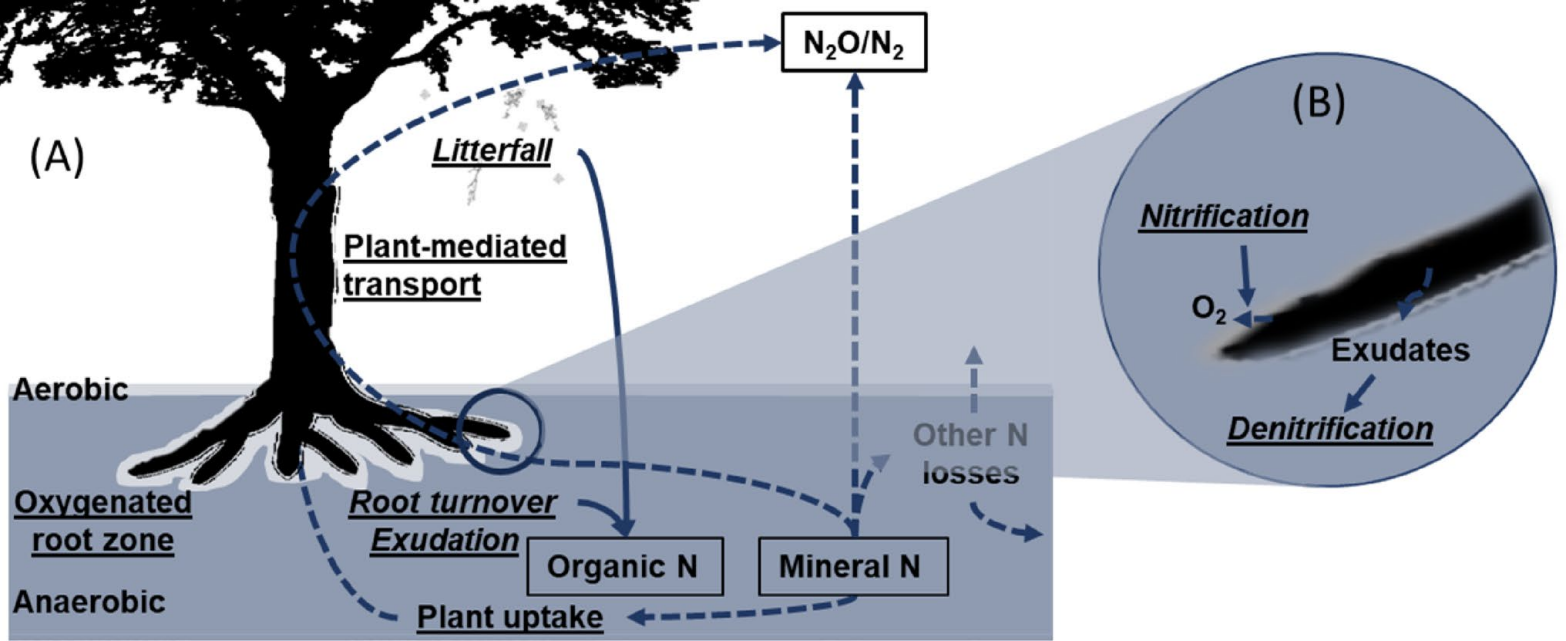
**A** Plant-mediated controls over tropical wetland N_2_O production, including plant-uptake of mineral nitrogen (N), plant litter inputs (leaf litterfall, root turnover), root exudation/rhizodeposition, plant-mediated transportation, and localised oxic zones around roots (**B**). Many key processes have been shown to be plant-species dependent

Collectively, these, and other nitrogen transformation pathways can be influenced by plants and their inputs via several mechanisms. These include plant nitrogen uptake, leaf, root and shoot inputs that determine soil and sediment biogeochemistry and thus decomposition (Wieder et al. 2011; Girkin et al. 2019) with root exudates regulating rhizosphere properties and representing an important substrate for denitrification (Girkin et al. 2018a, b). Root oxygen inputs control rhizosphere redox conditions (Girkin et al. 2020a, b), and plant vascular tissues act as a potential soil to atmosphere transport pathway for N_2_O produced in soils and sediments (Yamulki and Holt 2017) (Fig. 2). The interplay between these processes occurs within a spatially and temporally heterogeneous ecosystem and is further mediated by environmental variables including micro- and macro-topography, land use, and hydrology. As we discuss below, plants play a crucial role in shaping this heterogeneity, across all scales.

## Plant regulation of tropical wetland N_2_O emissions

Plants directly affect nitrogen availability in soils through root uptake of NH_4_^+^ and NO_3_^−^. In wetlands soils, nitrification can occur in surface soils under aerobic (non-flooded conditions) and close to roots that provide oxygen inputs (Fig. 2, Table 1) (Girkin et al. 2020a, b). However, wetland plants have often been considered to mainly take up NH_4_^+^, as NO_3_^−^ is often rapidly lost through denitrification. The precise balance of these processes varies between soil types, management and vegetation type (Kirk and Kronzucker 2005). In general, tropical climates feature distinct dry and wet seasons which can result in flooding pulses in wetlands (Liengaard et al. 2014). This remains important for plants, as lowered water tables will aerate soils, driving nitrification, and thereby affecting the forms of nitrogen available for uptake (Barrios and Herrera 1994).

**Table 1:** Processes by which plants may mediate N_2_O dynamics in tropical wetlands

Plant process	Impact(s)	Evidence from tropical wetlands	Indicative reference(s)
N uptake	Uptake of NH_4_^+^ or NO_3_^−^ can determine available N for nitrification–denitrification	Widespread evidence in model crops (e.g. rice)	Kirk and Kronzucker (2005)
Root exudation	Exudates provide substrates for denitrification	Evidence of diurnal trends in N_2_O, which may arise from changes in exudation, or temperature	Oktarita et al. (2017) and Teh et al. (2017)
Root oxygen loss	Creates localised oxygenated microsites within the rooting zone under otherwise flooded, low oxygen, conditions	No evidence of process in tropcial wetland plants; wider evidence available from other ecosystems	Philippot et al. (2009) and Girkin et al. (2020a, b)
Rhizosphere microbiome	Differences in microbial community structure between plant species	Limited evidence available for tropical wetland plants; wider evidence from other ecosystems	Espenberg et al. (2018) and Zhuang et al. (2020)
Plant-mediated N_2_O transport	Plant adaptations for root oxygenation (e.g. aerenchyma) support transport of GHGs from soils to the atmosphere	Limited evidence available for tropical wetland plants; wider evidence from other ecosystems	Jørgensen et al. (2012), Pangala et al. (2017) and Welch et al. (2019)
Photosynthesis	Direct production during photosynthesis	No evidence of process in tropical wetland plants; wider evidence from other ecosystems	Smart and Bloom (2001), Yamulki and Holt (2017) and Lenhart et al. (2019)

Plant litter inputs represent an important driver of soil N_2_O emissions, but precise effects vary based on litter properties and the environment in which decomposition occurs (Wieder et al. 2011). Seasonally flooded soils in the Amazon have previously been shown to be rich in inorganic nitrogen, although much is subsequently lost during water table drawdown (Koschorreck 2005). With the lowering of water tables, aquatic macrophytes can be left to decompose on draining wetland soils. In the Pantanal, Brazil, floating mats of *Pontederia crassipes*have been proposed to release 300–1000 kg N ha^−1^ yr^−1^, approximately 10 times as much as carbon (Koschorreck 2005; Sun et al. 2011). Phosphorus is often limiting in wetland soils (Cheesman et al. 2012), and low availability can limit the activity of nitrifying and denitrifying microbial communities (Yi et al. 2024). Combined, plant aboveground inputs therefore may represent a major seasonal driver of N_2_O emissions, equivalent to fertiliser applications in agroecosystems.

Rhizodeposition can both directly, through providing carbon substrates required for denitrification, and indirectly, by determining rhizosphere properties, affect N_2_O production. The largest component of rhizodeposition are root exudates, the composition of which depends on plant species, stage of development, soil properties, and prevailing environmental conditions (Badri and Vivanco 2009). This is important as the extent of denitrification is known to depend on both the quality and quantity of the carbon input, with labile sugars driving generally greater rates of denitrification than more complex organic molecules (Dodla et al. 2008). Root exudate profiles for most tropical wetland tree species are entirely unknown, but in general evidence suggests that organic acids are present in 2:1 or 3:1 ratios with sugars for many tree species, with different ratios reported for other plant functional types (Girkin et al. 2018a, b). Diurnal trends in N_2_O fluxes have previously been reported in wetland ecosystems (Teh et al. 2017), and may be due to increases in plant inputs of carbon derived from photosynthesis during daylight hours, or due to changes in temperature between night and day, but this latter contrast is reduced in tropical ecosystems compared to temperate latitudes (Jauhiainen et al. 2014; Girkin et al. 2025).

As well as being a substrate for the soil microbial community, litterfall, rhizodeposition and oxygen inputs can modify microbial community structure (Girkin et al. 2020a, b), and thereby indirectly regulate the extent of N_2_O production (Espenberg et al. 2018; Zhuang et al. 2020). The rhizosphere of wetland plant has previously been described as “oxic islands” which feature distinct microbial communities and diversity compared to bulk soils (Neori and Agami 2016). However, few studies have investigated the abundance and function of tropical wetland plant rhizosphere microbial communities beyond rice. In general, nitrogen depletion in the rhizosphere, through plant uptake or loss of nitrate, and the exudation of low nitrogen compounds can work alongside optimising pH and redox potential to promote nitrogen fixation (Husson 2012). The extent to which these processes differ between tropical wetland ecotypes, and different species, remains unclear.

Plant-mediated CH_4_ transport has been widely reported in tropical wetland ecosystems, resulting in stem and canopy emissions, thereby contributing substantially to ecosystem scale dynamics (Pangala et al. 2017), but N_2_O transport are less frequently assessed. Kreuzwieser et al. (2003) reported that the prop roots of *Rhizophora stylosa* emitted N_2_O at a rate of 3.3 µg m^−2^ root h^−1^. Studies in temperate forested wetlands (Yamulki and Holt 2017), and tropical dry forests (Welch et al. 2019) support the notion that some tree stems can be net N_2_O emitters but highlight that soil emissions tend to dominate ecosystem fluxes. N_2_O produced in the soil or dissolved in the porewater can be absorbed through the roots and transported through aerenchyma to aboveground tissues, where it is subsequently exchanged with the atmosphere. Evidence from studies of tropical wetland tree-emitted CH_4_ suggest this process is mediated by a range of aboveground adaptations, including lenticels, and prop and knee-roots, with high and low emitting species further differentiated through contrasts in root inputs (driving GHG production), aerenchyma volume, and wood density (Sjögersten et al. 2019).

N_2_O can also be produced during photosynthesis, from the reduction of NO_3_^−^, and during photo-assimilation of nitrite (NO_2_^−^) in chloroplasts (Smart and Bloom 2001). Upper estimates suggest this may account for 5–6% of total N_2_O emissions in agroecosystems, but there appears to be limited evidence of the importance of this process in wetland species (Yamulki and Holt 2017; Lenhart et al. 2019).

Plants can thus influence tropical wetland N_2_O dynamics through a wide range of processes. Given the diversity of wetland plant species, there is thus significant potential for species-specific differences in key processes (e.g. plant physiology, litter inputs etc.), to influence the extent of N_2_O emissions from different wetland ecotypes.

## Species-specific and ecotype controls

A limited number of studies in rice (*Oryza sativa*) have reported differences in N_2_O emissions between contrasting genotypes/varieties. For example, Baruah et al. (2010) reported significant differences in N_ N_2_O emissions between five rice varieties, identifying NO_3_^−^ availability as well as shoot and root mass, root length, and leaf area all as potential drivers of observed differences, with similar findings also reported by Slameto and Saputra (2024), and Zhang et al. (2024), amongst others. Lenhart et al. (2019) grew 32 plant species under controlled conditions to quantify the direct contribution of plants to N_2_O fluxes, reporting wide-variability across Genuses, but identifying a consistent strong correlation between N_2_O and CO_2_ fluxes.

However, few studies have assessed species-specific controls over N_2_O emissions in situ, while controlling for other important regulatory processes (e.g. degree of flooding, management, and soil properties) and often make use of multiple study sites with multiple differing characteristics, and studies that have assessed such processes often have small sample sizes, or are investigating processes using different vegetation types across multiple sites, thus confounding potential drivers. Yin et al. (2015) reported significant differences in N_2_O fluxes between three species (S*partina alterniflora*, an invasive perennial cordgrass, and *Suaeda salsa* and *Phragmites australis*), proposing that a negative correlation between N_2_O fluxes and aboveground biomass suggested differences in plant N uptake may have impacted available N for denitrification. In contrast, Were and Hein (2021) reported no significant differences in N_2_O emissions in the wet or dry season within a single Ugandan wetland site featuring *Typha latifolia, Phragmites mauritianus,* and *Cyperus papyrus.* Similarly*,* Marín-Muñiz et al. (2015) reported limited differences between marsh and swamps in coastal wetlands, as did Hernández and Junca-Gómez (2020) who found limited differences between dominant mangrove species, both in studies near Veracruz, Mexico. al. In contrast, Comer-Warner et al. (2022) reported significant differences in denitrification-derived N_2_O from *Melaleuca cajupti* forest soils compared to mangrove soils in Vietnam, but greater potential rates of total denitrification (N_2_O and N_2_) in mangroves (8.1 ng N g^−1^ h^−1^) than *Melaleuca* forest soils (6.8 ng N g^−1^ h^−1^). Such results may be because studied species exhibit relatively similar adaptations to their environment, and therefore the relative differences in species-specific controls are small, or the overriding importance of other environmental variables (e.g. WFPS) in these specific environments. Changes in environmental conditions (e.g. climate warming) are likely to impact fluxes directly (e.g. through increased microbial activity), but also through indirectly impacting plant regulatory processes, with such impacts again likely to be species specific in many wetland ecotypes to al.

### Impacts of global environmental change on tropical wetland N_2_O dynamics

Tropical wetland ecosystems, and plant processes, are already significantly affected by climate and land use change, with potential feedbacks for N_2_O dynamics (Table 2). Across all tropical ecosystems, temperatures are likely to increase (IPCC 2021), and more extreme weather events are predicted, including increased precipitation intensity (Endo et al. 2009), and more pronounced seasonality with lower precipitation during dry seasons but increases in wet seasons (Li et al. 2007). Collectively changes in precipitation will have significant consequences directly for N_2_O production, primarily by affecting WFPS (van Lent et al. 2015). Climate change is also likely to have a significant impact on tropical wetland ecosystem productivity through CO_2_ fertilisation effects impacting substrate availability, increased temperatures (also impacting the rate of microbial processes regulating N_2_O pathways) (Raturi et al. 2022), and drought/extreme flooding (Malhi et al. 2014). The impacts of this combination of processes have rarely been investigated in tropical wetlands compared to other ecosystems, and at present limited tools and models are available which can account for the relevant processes and predict likely responses (Farmer et al. 2011). In general, N_2_O emissions increase in line with primary productivity (Piñeiro-Guerra et al. 2019) of which temperature and moisture availability are major controls.

**Table 2:** Impacts of environmental change on tropical wetland N_2_O dynamics

Environmental change factor	Process impacted	Indicative reference(s)
*Climate change*		
CO_2_ fertilisation	Increased substrate availability through higher rates of ecosystem productivity	Raturi et al. (2022)
Temperature increases	Increased rates of plant litter and organic matter decomposition	Velthuis and Veraart (2022)
Precipitation changes	Increase or decrease in WFPS, impacting N availability for plant uptake	Liengaard et al. (2014) and Malhi et al. (2014)
*Management*		
Drainage	Increases N_2_O emissions through reduced inundation	Prananto et al. (2020)
Agricultural restoration	Decreases N_2_O emissions through species composition, and soil physicochemical properties	Comer-Warner et al. (2022)
Fertiliser applications	Increased substrate availability for denitrification	Zaman et al. (2008), Toma et al. (2011), Kachenchart et al. (2012) and Jovani-Sancho et al. (2023)
Liming	Changes in pH, impacting plant growth and N uptake	
Species/varietal changes	Shifts in composition alter balance of N flow pathways	Baruah et al. (2010), Slameto and Saputra (2024) and Zhang et al. (2024)

Land conversion has substantial consequences for GHG emissions from wetland ecosystems, by disturbing soils, changes in plant inputs, and alterations in management (van Lent et al. 2015). Drained peat soils can be a significant source of greenhouse gases (Girkin et al. 2023), including a substantial N_2_O source due to lower pH, which inhibits N_2_O reductase resulting in increased N_2_O as the end product of denitrification rather than N_2_ (Reddy and DeLaune 2008). Measurements of N_2_O fluxes from natural swamp forests can be up to 10 times lower than converted oil palm plantations due to plant fertiliser requirements and organic matter decomposition (Melling et al. 2007; Hergoualc’h and Verchot 2014; Oktarita et al. 2017). Similarly, Iram et al. (2021) highlight substantial changes in N_2_O emissions from land use change in coastal wetlands, with increased fluxes in drained pastures and adjacent sugarcane fields, particularly following fertilisation events (Iram et al. 2021). Castillo et al. (2017) demonstrated that N_2_O emissions from deforested mangrove areas were up to 34 times greater than those from intact forest.

## Conclusions

Tropical wetlands are a critical component of the global nitrogen cycle, representing a substantial organic nitrogen pool, but also a major source of N_2_O emissions. Many of the underlying mechanisms driving emissions remain unclear, including how alterations in fundamental ecosystem processes (changes in vegetation type and inputs, including litter and rhizodeposition) and shifts in management will interact with climate change to affect emissions. Collectively, this hampers the ability to generate a new generation of models for determining and upscaling dynamics. Growing evidence suggests a high potential for feedbacks, from both land use and climate change, giving an urgent need for quantifying remaining underlying mechanisms of regulation, and identifying potential pathways for mitigation for substantial tropical wetland N_2_O emissions.

## Data Availability

No new datasets were generated or analysed in this review.
